# Experimental resource pulses influence social-network dynamics and the potential for information flow in tool-using crows

**DOI:** 10.1038/ncomms8197

**Published:** 2015-11-03

**Authors:** James J. H. St Clair, Zackory T. Burns, Elaine M. Bettaney, Michael B. Morrissey, Brian Otis, Thomas B. Ryder, Robert C. Fleischer, Richard James, Christian Rutz

**Affiliations:** 1 Department of Zoology, University of Oxford, Oxford OX1 3PS, UK; 2 Department of Physics and Centre for Networks and Collective Behaviour, University of Bath, Bath BA2 7AY, UK; 3 School of Biology, Centre for Biological Diversity, University of St Andrews, St Andrews KY16 9TH, UK; 4 Department of Electrical Engineering, University of Washington, Seattle, Washington 98195-3770, USA; 5 Smithsonian Conservation Biology Institute, National Zoological Park, Washington, DC 20013-7012, USA

## Abstract

Social-network dynamics have profound consequences for biological processes such as information flow, but are notoriously difficult to measure in the wild. We used novel transceiver technology to chart association patterns across 19 days in a wild population of the New Caledonian crow—a tool-using species that may socially learn, and culturally accumulate, tool-related information. To examine the causes and consequences of changing network topology, we manipulated the environmental availability of the crows' preferred tool-extracted prey, and simulated, *in silico*, the diffusion of information across field-recorded time-ordered networks. Here we show that network structure responds quickly to environmental change and that novel information can potentially spread rapidly within multi-family communities, especially when tool-use opportunities are plentiful. At the same time, we report surprisingly limited social contact between neighbouring crow communities. Such scale dependence in information-flow dynamics is likely to influence the evolution and maintenance of material cultures.

## Abstract (web summary)

Social-network dynamics can influence biological processes such as information flow, but are difficult to measure in the wild. Here, St Clair *et al.* track networks of New Caledonian crows to show how the potential for information flow can be influenced by changes in resource distribution.

## Graphical abstract

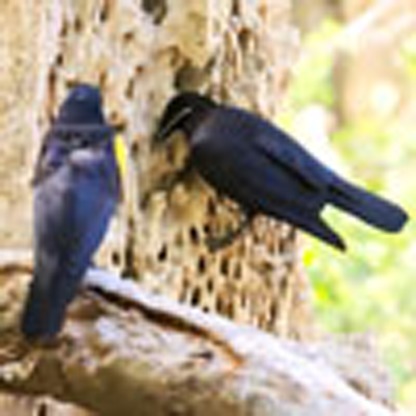

New Caledonian crows *Corvus moneduloides* (hereafter ‘NC crows' or ‘crows') use a range of twig and leaf tool types to extract embedded prey^1^ (Fig. 1a,b). Earlier research has highlighted the possibility that NC crows may socially learn at least some of their tool-related skills, leading to increasingly complex tool technologies and geographically distinct material ‘cultures', but this has not been formally tested^2^,^3^,^4^. The emergence and maintenance of cultural variation depend not only on the mode of social transmission, but also on the dynamic structure of the network across which information flows^5^,^6^,^7^,^8^. This is because the sequence, frequency and proximity of social encounters determine which individuals are in a position to transmit or learn information at any given time and, by extension, the speed and pathways of diffusion^5^. It is extremely challenging, however, to collect such fine-scale, time-ordered data from free-ranging animal populations, especially in inaccessible habitats^9^,^10^. We used a ‘reality mining' approach^10^,^11^,^12^ to record associations in a wild NC crow population, combining cutting-edge tracking technology, large-scale field experiments and computer simulations.

Using self-developed wireless sensor network (WSN) technology (‘Encounternet'^13^,^14^), we charted association patterns and space use of a population of 33 free-ranging NC crows, of known genetic relatedness, during a 19-day study period. Birds were fitted with miniature (*ca.* 10 g) transceiver tags that transmitted ID-coded radio pulses every 20 s, while receiving and logging signals from other nearby tags, producing detailed records of the time, proximity and duration of crow encounters. Meanwhile, an array of 45 fixed receivers (‘basestations'; Fig. 1c) monitored roaming tags, and wirelessly downloaded their logged association data. This technology enabled us to map social-network dynamics: directly (that is, encounters were not inferred *post hoc* from group membership data); continuously (in contrast to some other technologies^15^, logging of encounters can occur when subjects are out of range of fixed receivers); remotely and autonomously (eliminating disturbance by human observers); at unprecedented temporal resolution (in the order of minutes); over a biologically relevant range of bird-to-bird distances (in the order of metres); and in near real time (each day's association data could be retrieved and analysed overnight).

The distributions of animals in space, and thus their possible association networks, are likely to be strongly affected by the distribution of key resources^9^,^16^,^17^. To examine the influence of changes in local resource abundance on network dynamics, we manipulated the environmental availability of an important crow food source, following a simple baseline–experiment–reversal design. Specifically, we mimicked natural resource gluts that occur when decaying candlenut trees *Aleurites moluccana* fall and break open (‘tree-fall events'; Fig. 1a), exposing the burrows of highly nutritious longhorn beetle larvae *Agrianome fairmairei*, which crows dexterously extract with stick tools^18^,^19^,^20^ (Fig. 1b). Finally, to examine the potential consequences of the observed network dynamics for the spread of information by social learning, we used our empirical association data to simulate the diffusion of information across networks recorded under these different conditions. Our analyses reveal that the potential for information flow responds rapidly and heterogeneously to changes in resource availability—dynamics that are likely to influence the capacity of crow networks to support cultural variation.

## Results

### Structure of the undisturbed social network

**Figure 1:**
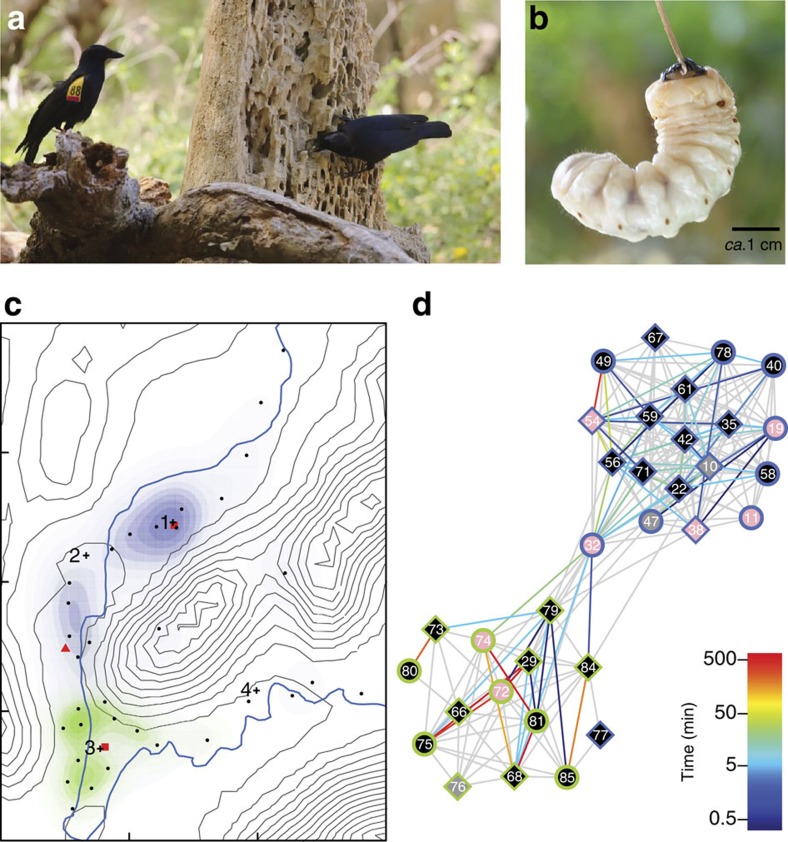
Space use and association network of a wild, undisturbed population of New Caledonian crows. (**a**) A wing-tagged crow observes another individual using a stick tool at a natural tree-fall site, where the burrows of longhorn beetle larvae have become exposed (photo: J. Troscianko). (**b**) A beetle larva (*ca*. 60 mm in length) bites the tip of a researcher-held ‘tool'. (**c**) Topological map of the study site (10 m elevation contours; 1,000 m grid), showing the location of crow traps (numbered crosses; traps were removed before the study), fixed receivers (‘basestations'; filled circles) and experimental ‘tree falls' during E1 (red triangle) and E2 (red squares) periods. Spatial segregation of crow communities, as inferred from tag signals received by basestations over the baseline period, is shown by kernel density plots (blue plot based on pulses from birds in the northern community; green plot for the southern community). (**d**) Association network of both crow communities over the same time period. Short-range associations are shown in colours corresponding to the time (in minutes) spent in association (note logged scale), while more distant associations are shown in uniform grey. Sex is indicated by node shape (female, circle; male, square), age by node colour (juvenile, pink; immature, grey; adult, black) and trap location by node boundary colour (northern community, blue; southern community, green).

Our system yielded a total of 177,102 daytime association logs. Unless stated otherwise, our analyses are based on a subsample of logs with a high mean signal strength (*n*=4,268 logs of RSSI*mean*≥15, classified as 1,820 discrete encounters), representing encounters during which crows are likely to have been close enough to observe each other's tool-oriented behaviour (see Methods). A static (time-aggregated) network for the initial 7-day perturbation-free ‘baseline' period (‘B') exhibited clear assortment by capture site (Fig. 1c,d; Newman's assortativity *r*=0.8318; σ_*r*_=0.042)^21^, evidently forming a northern community (20 birds) and a smaller southern one (13 birds). Tag pulses received by the basestation array confirmed that community membership and space use were strongly linked (Fig. 1c). Association patterns during each baseline day were significantly correlated with genetic relatedness because of a tendency for first-order related dyads to spend more time in close proximity than more distantly related ones (daily Pearson correlations: *r* ranging from 0.415 to 0.564, *n*=7 days, with all *P*<0.03)^22^. Each community contained several well-connected dyads or triads, usually comprising genetically unrelated adult male–female dyads (presumably bonded pairs), and/or related adult–juvenile pairs^13^.

### Effects of experimental resource provisioning

**Figure 2:**
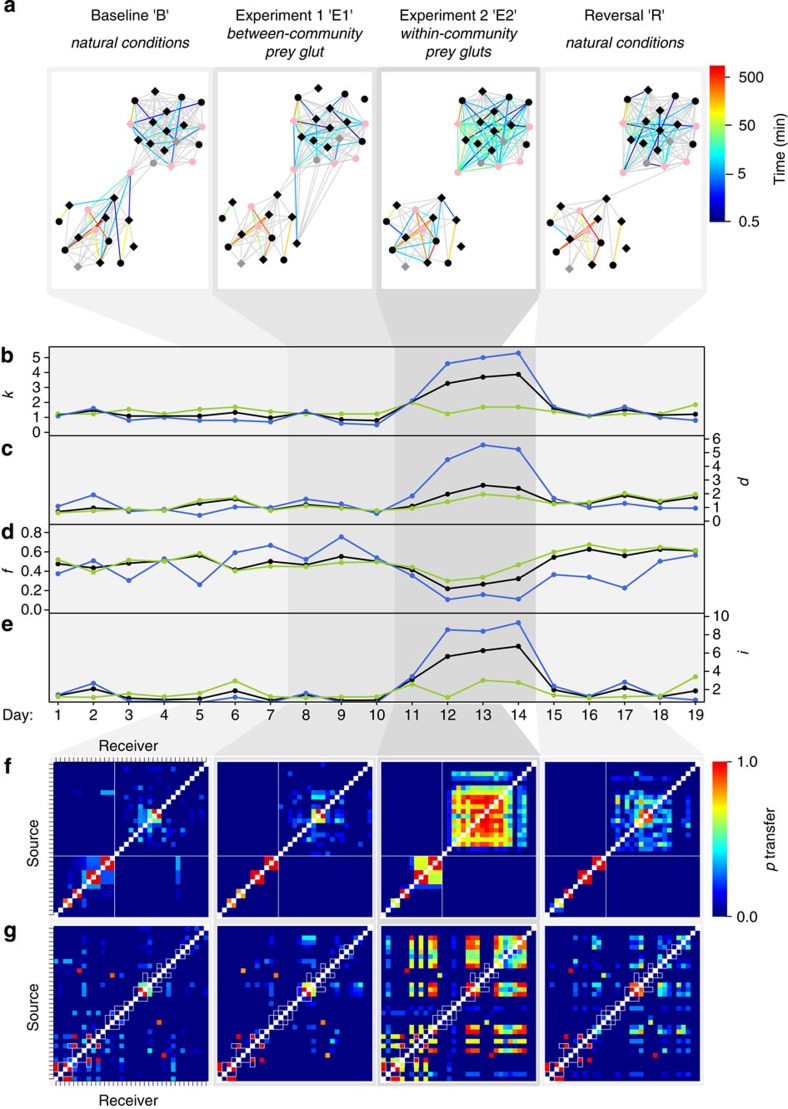
Effects of experimentally manipulated tool-use opportunities on the structure, dynamics and information-flow potential of a social network of wild New Caledonian crows. (**a**) Association networks based on 3-day subsamples (for visual comparison) for all four experimental periods. (**b**) The mean degree (*k*) of daily networks for the whole population (black), and separately, for the northern (blue) and southern community (green) only. (**c**) The mean of the total time spent in close association (*d*) by individual crows on each day, scaled to baseline levels for ease of comparison. (**d**) Proportion of time spent in close association that was with first-order genetic relatives (*f*). (**e**) The mean daily indomain size (*i*) from simulations of social information flow across dynamic networks. (**f**,**g**) Matrices summarizing information-flow simulations on time-ordered 3-day association networks (same as in **a**). Cell colours correspond to the probability that information passes from one crow (arranged along the *y* axis) to another (*x* axis) during each experimental period. Nodes are ordered according to either community membership (**f**), or genetic relatedness (**g**); in (**g**), cells corresponding to 1st-order related individuals are enclosed in white boxes.

Nightly retrieval and analysis of association data allowed us to confirm that the network was in a stable state before experimental manipulations commenced (Fig. 2a–c). We first recreated a tree-fall event at a position approximately midway between the core regions occupied by the northern and southern communities (Fig. 1c). Interestingly, this experiment (‘E1') failed to induce significant topological changes in daily time-aggregated networks during its 3-day duration (Fig. 2a–c). Tag signals logged by a nearby basestation revealed that two subjects approached the experimental site to within *ca.* 10 m, but neither bird landed on the provided logs, and no other tagged birds were recruited, highlighting a surprising degree of community segregation (Fig. 1c,d). In contrast, a second experiment (‘E2') of community-central tree falls (Supplementary Fig. 1), implemented following reversal of E1, elicited strong responses at both within-community and whole-network levels. Node-based metrics—mean degree (*k*) and total time spent in associations (*d*)—increased markedly (over three- and twofold, respectively) during the 4-day E2 experimental period, relative to preceding periods [*k*=1.09 (range: 0.97–1.45) for the seven B days versus 3.48 (2.06–3.88) for the four E2 days, Wilcoxon rank-sum test *W*=0, *P*=0.010, estimated difference=2.26, 95% confidence interval (CI)=0.97–2.79; *d*=0.89 min (0.73–1.71) for the seven B days versus 2.31 min (1.16–2.77) for the four E2 days, Wilcoxon rank-sum test *W*=2, *P*=0.024, estimated difference=1.21, 95% CI=0.26–1.88], suggesting that a novel network state had been achieved (Fig. 2a–c).

Experimental resource provision during E2 increased network connectivity in two ways. First, within each community and across the population as a whole, novel edges formed among hitherto non-associating birds (54 novel dyadic associations occurred population-wide during E2, beyond the 73 recorded during the preceding 10 days; Supplementary Fig. 2). Second, dyads that had previously associated tended to increase their association time. These processes occurred differentially in the two communities: the predominant response in the northern community was the formation of new associations, while the southern one responded largely with the strengthening of existing bonds (Fig. 2b,c). At the population level, these changes produced a significant reduction in genetic assortativity, with values of *f*—the proportion of time spent in close encounters that was with first-order kin—falling to a median of 0.31 (0.219–0.414) during the four E2 days, from 0.48 (0.415–0.564) during the 7 days of period B (Wilcoxon rank-sum test *W*=28, *P*=0.006, estimated difference=0.18, 95% CI=0.07–0.28; Fig. 2d). Taken together, these results imply a marked increase in the number and strength of horizontal and oblique transmission pathways, across which tool-related and other social information could potentially ‘jump' between non-family lineages.

While the aggregation of association data into a series of static networks allows convenient comparison of network properties within and between different experimental periods, this ‘snapshot' approach can mask important biological patterns, such as how information might spread^23^,^24^. We were able to examine dynamic changes in crow association patterns minute-by-minute (Supplementary Fig. 3). These fine-scale analyses revealed that our population's response to the E2 resource glut was largely due to brief (*ca.* 1 h long) periods of intense early-morning association (in contrast to B and E1 days), consistent with aggregation at resource patches and dispersal following satiation or prey depletion. This lack of effective resource defence by dominant birds, and apparently high level of tolerance to unrelated conspecifics at prime foraging sites, is unexpected and contrasts with the results of an earlier study that tracked NC crows sporadically by means of conventional radio-telemetry^25^. In primate societies, a similarly ‘egalitarian' social context of tool-assisted foraging provides abundant opportunities for social learning, through observation, co-action or interaction with freshly discarded tools^26^, and is thought to be key to maintaining and accumulating cultural information^27^,^28^,^29^.

### Simulated information flow on time-ordered networks

To explore how our observed dynamic networks could support information flow, we implemented simulations following the basic rationale of ‘susceptible infectious' compartment models of disease transmission^30^. In each simulation run, one crow was chosen to be the source of a novel piece of information at the start of a day; all others were naive. Information could only spread from an informed to a naive individual if they had a close-range encounter on that day. All informed crows remained informed, and could therefore act as potential spreaders, for the rest of the day. At the end of the day, informed crows were listed. In subsequent runs, each crow in turn was chosen as the ‘seed' for the daily information flow, which again could only occur during the observed encounters between crows on the chosen day. To measure simulated information spread during a day, we counted the number of seeds from which each crow could have received information (that is, its ‘indomain'), which gives similar counts to the ‘outdomain', the number of crows ultimately informed by each seed. As information transfer was modelled stochastically (see Methods), we repeated the simulation 1,000 times for each seed on each day.

As all empirically recorded daily networks contained both strongly connected individuals and more weakly connected or isolated ones, it follows that indomains (and outdomains) should vary according to which crow was the seed individual on a given day. We did indeed find substantial variation in our simulations: for example, depending on the seed chosen, outdomains averaged over B days ranged from 1 to 3.7 crows, and from 1 to 11.5 crows for E2 days. To allow conclusions about network properties across all nodes, we averaged indomain size over all crows and replicates to produce the measure *i*, the mean indomain size of a given group of crows on a given day, which serves as a proxy for the information-carrying capacity of the observed network. As might be expected, *i* was sensitive to the rate of information transfer within encounters (see Methods), but varied little between days during the baseline (B) and the first experimental period (E1) (Fig. 2e). Critically, the mean indomain size increased dramatically in response to E2 tree-fall events, with crows in a position to receive information from over five times as many possible seed crows on E2 days than on B days [Fig. 2e–g; median *i*=5.95 (3.09–6.74) for the four E2 days versus 1.05 (0.83–2.08) for the seven B days, Wilcoxon rank-sum test *W*=0, *P*=0.006, estimated difference=4.65, 95% CI=2.05–5.70]. These findings suggest that variation in resource distribution is a key driver of the information-flow properties of NC crow social networks.

### Reversibility of experimental effects

Following removal of E2 tree-fall stimuli after four days, we continued to monitor network topology for five more days to probe the persistence of experimental effects. In this perturbation-free reversal period (‘R'), most aspects of network structure tended to return to baseline levels (Fig. 2). This pattern of reversal supports the causal effect of our E2 experiment on network topology and illustrates the speed with which association networks can track changing environmental conditions. Some metrics during the reversal period differed from their pre-manipulation levels [*d*=1.46 min (1.36–1.99) for the five R days versus 0.89 min (0.73–1.71) for the seven B days, Wilcoxon rank-sum test *W*=4, *P*=0.030, estimated difference=0.57, 95% CI=0.08–1.03; *f*=0.61 (0.544–0.627) for the five R days versus 0.483 (0.414–0.564) for the seven B days, Wilcoxon rank-sum test *W*=2, *P*=0.010, estimated difference=0.12, 95% CI=0.05–0.18; Fig. 2, Supplementary Fig. 7]. Although the difference in the mean duration *d* between B and R could be due to temporal autocorrelation between experimental and post-experimental periods, we note that such a ‘lag' did not prevent the rapid expression of experimental effects during E2, and that *f* (the proportion of time in association that was spent with kin) was actually significantly higher during R than during any previous period. Taken together, these findings imply that some behavioural drivers of network topology may be sensitive to both the current and the past distribution of prey resources.

## Discussion

Association network dynamics are profoundly linked to evolutionary and ecological processes through effects on predation risk^31^, disease transmission^24^,^32^, reproductive opportunities^33^, costs and benefits of cooperative behaviour^34^, and (as here) the opportunity for social information exchange^8^,^15^,^34^. Until now, such dynamics have been extremely difficult to investigate in wild animal populations. Our study demonstrates that, while NC crow social structure is influenced by genetic relatedness, close and protracted associations between unrelated birds provide abundant opportunities for social information exchange between lineages. Major experimentally-induced increases in information-flow potential suggest that clumped prey resources, as are known to occur naturally when beetle-infested trees topple and break open^18^,^20^ (Fig. 1a), may accelerate the diffusion of tool-related knowledge in NC crow communities.

It is worth stressing that changes in whole-network properties were recorded within hours of both the start and end of the E2 period, a timescale that would have been impossible to resolve using alternative methods of observation^10^. We expect that advances in WSN technology will allow the collection of ever larger, richer and better-resolved social-network datasets, from a growing range of study animals and environments. As we have shown, such methods facilitate experimental manipulation of entire social networks of free-ranging animals and allow responses to be recorded with unprecedented temporal resolution. Obvious applications include further field-experimental investigations of the environmental causes, and functional consequences, of social-network dynamics. Importantly, our simulation-based approach allows emergent properties such as information-flow potential to be integrated across repeat runs with all possible ‘seed' individuals, averaging out stochastic and inter-individual variation. This method should complement traditional field-diffusion experiments, which track the effects of ‘one-shot' seeding of novel information by training a small number of pre-selected demonstrators as a critical test of diffusion by social learning^35^. Our findings should inform the design and interpretation of such field-diffusion studies, which often use localized food sources as opportunities for subjects to express and (socially) learn novel behaviours, or to attract individuals in order to infer social bonds between them^35^. In both cases, estimates of the probability, speed and routes of information exchange may be strongly influenced by experimentally elicited levels of aggregation that may, for some study species, be highly unusual.

In NC crows, naturalistic tree-fall manipulations markedly increased the potential for information flow within communities, while at the same time we found very low rates of association between communities centred only a few hundred metres apart (Figs 1c,d and 2f). This pattern suggests a modular social structure^6^ in which between-community information flow is likely to occur over disproportionately slow timescales, mediated by population-dynamic processes such as natal or breeding dispersal^36^. Empirical studies suggest that reduced information flow between social groups can facilitate cultural divergence^37^,^38^, in much the same way that restricted gene flow between populations permits genetic divergence. It is thus possible that the observed scale dependence in information-flow dynamics facilitates the evolution and maintenance of tool-related variation.

## Methods

### Study site and subject attributes

We conducted our study from 2 October to 15 November 2011 in a lowland dry-forest study site on the central west coast of Grand Terre island, New Caledonia (Gouaro-Déva, 21°33′40′′S, 165°19′00′′E; Fig. 1c). The resident crow population had been undisturbed by research activity^4^,^18^,^39^ for *ca.* 2 years before the current study. From 2 to 21 October, 42 crows were trapped using meat-baited whoosh nets^40^, deployed at four different locations (Fig. 1c). At two sites, capture rates diminished over the course of trapping, while the other two sites produced only one crow each (Supplementary Fig. 5). We estimate that these 42 birds constitute over 80% of the local crow population during our study period. We note that these crows are likely to be a nonrandom sample, as neophobic individuals that were wary of approaching trapsites (and may also have avoided our experimental manipulations) are less likely to have been included. All trapped crows were blood-sampled (for molecular sexing^41^), allocated to age categories (according to gape colouration, which changes as individuals mature^20^,^42^), weighed, measured and tagged, with the exception of an adult male, which had an injured wing and was released immediately. We genotyped individuals at 12 polymorphic loci^36^ and used Kinship to generate a binarized matrix G of first-order relatedness^43^,^44^. Following best-practice guidelines for biologging studies^45^, backpack-mounted tags (including braided nylon weak-link harnesses; Sirtrack, NZ) totalled less than 5% of the recipient's body mass in all cases^13^. For summary information on subjects and their tags, see Supplementary Table 1.

### System overview

‘Encounternet' is a fully digital animal-tracking system that uses advanced WSN technology; a detailed description of hardware components and basic functionality has been provided elsewhere^13^,^14^. Briefly, in this first full-scale deployment of a proximity-enabled Encounternet system, crow-mounted transceiver tags were programmed to emit regular ID-coded radio pulses (see ‘Software settings'), while recording pulses from all other tags within reception range. Tag-to-tag encounters were written to ‘logs', which were stored in the on-board memory of each participating tag. Logs included the identities of the sending and receiving tags, time data from on-board clocks and a ‘received signal strength indicator' (RSSI) summary of received radio pulses, which allowed *post hoc* inference about the proximity of associating birds (see ‘Estimating bird-to-bird distance from signal strength'). When a tag containing stored logs came within communication range of a fixed receiver (‘basestation'), 45 of which were arrayed across our study site (Fig. 1c), all logs were transferred wirelessly from tag to basestation, and automatically cleared from tag memory following successful upload. Basestations also directly recorded the time and sender-ID of all radio pulses from tags within reception range, providing useful spatial context for the association dataset (Fig. 1c). Finally, hand-held transceivers (‘masternodes') were used by fieldworkers to wirelessly download logs from tags and basestations, and to perform software adjustments, such as routine synchronization of the on-board clocks of all tags.

### Basestation array and tag detection

We deployed a higher density of basestations in areas of preferred crow habitat, in order to maximize data retrieval from roaming tags (Fig. 1c). At night (see ‘Experimental schedule') we visited each basestation in turn, using masternodes to download the encounter logs accumulated during the preceding daylight period. Basestations in areas of high crow density typically recorded thousands of logs over a 24 h period, while units positioned at the far north and south of the main study valley acquired few (if any) data, suggesting that the array was appropriately widespread to monitor our study population^13^. The maximum number of encounter logs recorded on any crow-borne tag was 885, while the maximum number recorded on any basestation was 58,645, substantially below the memory capacity of tags and basestations respectively, implying that our system was never memory-limited. Every night before programmed tag-shutdown at 20:00 (starting no earlier than 18:45; see ‘Software settings'), we remotely contacted tags on roosting birds using masternodes, to ensure clock synchronization. Of our 41 deployed tags, four apparently failed to transmit, as they were never detected by other tags, basestations or masternodes, even during regular, wide-ranging searches outside the core study area. A further four tags either ceased transmitting, or developed irremediable on-board clock issues during the study (on average 8.0±1.6 days after data collection had commenced), and were excluded from all analyses. Of the remaining 33 tags, 26 delivered immediately useable data, while the on-board clocks of the other seven had drifted, or spontaneously reset at least once during deployment, which necessitated *post hoc* adjustment of logged times (see ‘Data processing').

### Experimental schedule

From the time that traps were permanently removed from the study area (21 October 2011) until data collection ceased, fieldworkers only entered the study site during the crows' inactive roosting period (range: 18:45–05:00) to ensure that the social network under investigation was minimally disturbed. Tags switched on synchronously at 04:00 on 27 October 2011 (see ‘Software settings'), after a 5-day standby period during which the birds could habituate to their tags, and the population could recover from our trapping activities. After initial switch-on, tags operated on a 16/8 h on–off duty cycle (see ‘Software settings' below). Data were collected over 19 consecutive days, divided into four experimental time periods: a 7-day perturbation-free ‘baseline' control period (‘B'); a 3-day experimental period with a simulated tree fall in the centre of the main study valley, approximately midway between the two crow communities (‘E1'); a 4-day experimental period with a simulated tree fall close to the geographical centre of each crow community (‘E2'); and a 5-day post-experimental reversal period (‘R'), replicating baseline conditions. Each tree-fall simulation (E1, 1 site; E2, 2 sites; Fig. 1c) consisted of *ca.* 150 kg of decaying candlenut timber transported to the experimental sites and collected into a pile as though an infested tree had freshly fallen^46^ (Supplementary Fig. 1). This timber was ‘salted' each night with longhorn beetle larvae, which were collected elsewhere and placed carefully into existing beetle burrows and crevices^19^. For logistical reasons, the E1 stimulus was covered with a tarpaulin at the end of the E1 period, while both E2 stimuli were physically removed from the study site at the end of the E2 period. As with all data collection and system maintenance, experimental stimuli were positioned and removed at night, to avoid crow disturbance.

### Software settings

We programmed tags to optimize system performance within the constraints imposed by their memory and battery capacity. Following the initial standby period (see above), tags operated on a 16/8 h on–off duty cycle, starting at 04:00 each morning (68.4±0.7 min before sunrise) and shutting down every evening at 20:00 (112.1±0.7 min after sunset). Tags were programmed to emit a single radio pulse (433 MHz) every 20 s. Since on-board memory may have been insufficient to record every single received radio pulse individually, tags were programmed to dynamically calculate the mean received signal strength (RSSI*mean*) for pulse sequences during sustained encounters, and to record this value in a log, together with the identities of transmitting and receiving tags, the start- and stop-time of the pulse sequence (T*start* and T*stop*), and the power of the weakest (RSSI*min*) and strongest (RSSI*max*) received radio pulses. The receiving tag closed the log when either (*i*) no pulse was received from the transmitting tag for six consecutive pulse intervals (in our study, 6 × 20 s=120 s); or (*ii*) the encounter reached a threshold of 15 received pulses (in either case, T*stop* would equal the time of the last received pulse). For encounters that exceeded 15 pulses, fresh logs were initiated sequentially for as long as the encounter continued (for example, a 12-min encounter would generate a series of three separate logs, of 5, 5 and 2 min, respectively). Condition (*i*) above was designed to allow for accidentally missed pulses, occurring (for example) because of temporary obstructions between transmitting and receiving tags. Condition (*ii*) was implemented to reduce information loss through averaging over the course of protracted encounters and caused logs to record a maximum duration (T*stop* minus T*start*) of 300 s in the case that 15 consecutive 20-s pulses were received. As condition (*i*) effectively allowed six consecutive missed pulses during the course of an encounter, however, it was possible for encounter logs to last for longer than 300 s^14^.

### Estimating bird-to-bird distance from signal strength

‘Proximity loggers', including our Encounternet transceiver tags, record the strength of received radio signals (RSSI; see above), from which the distance between tags (and hence animals) is later estimated. Such inference is possible because radio signals attenuate predictably with distance from their source. Before deploying our system, we experimentally explored the effects of distance and several nuisance variables (habitat structure; height of tags above ground; relative alignment of transmitting and receiving tag antennae) on RSSI (reported in full elsewhere^14^). The resulting statistical model allowed us—given some assumptions about the movement of crows (for example, that relative antenna angles were sampled randomly) and their habitat preferences and activity profiles (as characterized in previous years with crow-mounted, miniature video cameras^47^)—to establish an RSSI threshold value, with which we could identify relatively close encounters, during which crows may have observed each other. While RSSI*max* (see above) provides a convenient tool for preliminary analyses^13^, high values may be generated by fleeting passes of birds, rather than by protracted social encounters. To address this problem, we filtered our raw dataset using RSSI*mean* values instead, which are computed by averaging RSSI across series of consecutively received pulses (see above) and therefore contain considerably more information. This averaging process has two noteworthy consequences. First, the RSSI*mean* of many encounters (in particular, very short ones) will be reduced by weaker pulses during the approach and separation periods of associating individuals; therefore, we will have discarded a number of encounter segments in which crow pairs came within close proximity at least part of the time. Second, because animals are unlikely to be stationary throughout an encounter, their distance of closest approach will generally be much lower than the recorded RSSI*mean* suggests. To obtain a dataset for our network and diffusion analyses comprising predominantly of close encounters, during which social learning through close observation could have taken place, we applied a filtering threshold of RSSI*mean*≥15. Our calibration analyses suggest that a single-pulse RSSI of 15 will correspond to an inter-crow distance of ≤4.7 m in 50% of cases, and ≤11.3 m in 95% of cases^14^. These distance estimates are likely to be highly conservative: of all logs with RSSI*mean*≥15, we found that the median RSSI*max* value was 37, indicating that dyads generally spent part of their encounter in much closer proximity than the RSSI*mean* value suggests.

### Data processing

Before analysis, we conducted a quality-control exercise^48^, in which: (*i*) incomplete, corrupt or duplicate logs were removed; (*ii*) data from tags that failed temporarily or wholly during the 19-day deployment period were discarded; (*iii*) the timing of encounter logs was compared visually between each tag in a given dyad, to identify offsets that might indicate clock-drift issues; and (*iv*) *post hoc* time adjustments were made to logs of seven tags with faulty clocks. Step (*iii*) was omitted for one tag (ID no. 35), which was actively transmitting throughout the deployment, but which failed to upload any logs to basestations, so that its encounters were recorded only by its social partners' tags rather than by both participating tags. Time adjustments in step (*iv*) were based on tag clock readings, which we had recorded from each tag at known times using the masternode on an approximately nightly basis (see above). These adjustments were also facilitated by the basestation array, which recorded the activity schedule of the affected tags, as well as by unaffected tags, which reliably logged the time of encounters with affected tags. For social-network analyses and graphs, we subsampled tag-to-tag logs according to their RSSI*mean* values in order to include only encounter segments where crows remained within a given distance of each other on average (see above; Supplementary Fig. 6a,b). Because each encounter between tagged birds is effectively recorded twice (once by each participating tag), it is necessary to reconcile between-tag discrepancies in RSSI and start- and stop times. Such discrepancies can arise from phase offsets in pulse patterns between tags, from minor clock asynchronies, and from between-tag power differences likely associated with small variations in power cells, amplifier circuits and antennae^14^,^49^; we reconciled such log pairs by accepting the earliest start time and the latest stop time. Finally, because protracted encounters generated a series of *ca.* 5-min long logs (see above), we considered logs that started within 23 s of each other (marginally over a single-pulse interval; see above) to be contiguous and amalgamated them to form a single encounter record^50^ (Supplementary Fig. 6c).

### Network analyses

Node layout for network graphs was the same in all figures and was achieved by spring-embedding, with minor manual adjustments to improve legibility. ‘Close' associations are indicated by edges coloured according to the summed duration of associations with an RSSI*mean*≥15 (see above) logged during the period of interest (Fig. 1d, 7 days; Fig. 2a, 3-day subsamples to allow comparison). More distant spatial associations (RSSI*mean*≥0) are indicated by grey edges. To produce time-series plots (Fig. 2b–d), we used daily aggregated associations, stored in a matrix D of the total (daily) duration of pairwise encounters with RSSI*mean*≥15 (see above). We computed each metric (*k*, *d* and *f,* see below) on each of the 19 consecutive study days, first for all 33 crows, and then separately for the northern and southern communities (here each community was treated as an ‘isolated' group with its own duration matrix D). The degree of a crow on a given day is the number of other tagged crows it encountered (at the specified RSSI*mean* level); for crow j, it is simply the count of non-zero elements in row j of matrix D. This number, averaged over all crows (*n*=33, 20 or 13), gives the mean daily degree (*k*). The mean duration of encounters (*d*) is the average, over all possible crow dyads, of the daily duration of encounters, computed as the average of the off-diagonal elements of D. Encounter durations among crows in the southern community were substantially longer than in the northern one on average; therefore, for the purposes of visualization (Fig. 2c), the mean duration was scaled by the average value in the first seven baseline days (1.07 min for all crows, 0.55 min for the northern and 5.79 min for the southern community). Molecular analysis of crows' blood samples (see ‘Study site and subject attributes') generated a binarized matrix G of first-order relatedness^36^. We used two approaches to explore the role of relatedness in shaping daily association networks. The first was to correlate the association matrix for all crows for each day of the baseline period (days 1–7) with matrix G. We used standard Mantel matrix correlation tests for this, with the Pearson correlation coefficient as the test statistic. To test the hypothesis that the correlation coefficient was greater than expected by chance, we used a stratified node permutation test in which node labels of crows from the northern community (and from the southern community) were shuffled only among themselves^51^. The second approach (shown in Fig. 2d) examined changes in the fraction of daily encounter durations that occurred between pairs of first-order related crows (*f*). If all non-zero entries in matrix D align with non-zero entries in G, *f*=1.

### Information-flow simulations

The potential flow of social information across networks was investigated through a series of daily simulations (Fig. 2e–g; for details, see main text). In each simulation, a single crow was seeded with a hypothetical piece of information at the start of the day. The information could pass from ‘informed' to ‘naive' crows during encounters between them, simulating the spread of information through the population. Within the time window of each encounter, the transmission process was modelled to be stochastic, with a constant probability per unit time, characterized by the mean time for information transfer (*λ*). We set *λ* at 5 min, reflecting the modal duration of empirically observed encounters. Each crow in turn was chosen as the information seed. Our measure of information flow is the size of the ‘indomain'; for crow j, this is the number of seed crows from which crow j could have received the hypothetical piece of information by day's end. We ran 1,000 simulations per starting crow per day and plotted the daily indomain size averaged over the 1,000 simulations and over all crows, those in the northern community, and those in the southern one (*i*; Fig. 2e). Varying *λ* by±3 min caused the average all-crow indomain (*i*) to vary in size by less than one crow. For more detailed analyses of indomain effects (Fig. 2f,g), we plotted matrices that summarize data from the same contiguous 3-day blocks (B, E1, E2 and R) that had been used to construct network graphs (Fig. 2a). Each cell in the matrix panels was coloured according to the probability of simulated information passing from the starting crow (arranged along the *y* axis) to another crow (*x* axis), averaged over all runs on all days used in the relevant 3-day period (the only difference between panels f and g is the ordering of the crows; for details, see figure caption). Wilcoxon rank-sum tests were used to compare values of daily network metrics between experimental time periods; to illustrate effects, the medians are presented, together with the full data range, and the estimated effect size (the median of the difference between a sample from the first group and a sample from the second) with its 95% confidence interval. Finally, because the maximum distance at which individuals are considered to be ‘in association' will influence the results of social-network analyses, we generated descriptive metrics for each experimental period using a range of RSSI*mean* threshold values (RSSI*mean*≥13, 14, 16 and 17), demonstrating that the described results remained qualitatively unchanged (Supplementary Fig. 7).

## Additional information

**How to cite this article:** St Clair, J. J. H. *et al.* Experimental resource pulses influence social-network dynamics and the potential for information flow in tool-using crows. *Nat. Commun.* 6:7197 doi: 10.1038/ncomms8197 (2015).

## Supplementary Material

Supplementary Information Supplementary Figures 1-7, Supplementary Table 1 and Supplementary References.
