# Supplementary material for: Experimental resource pulses influence social-network dynamics and the potential for information flow in tool-using crows
Source: Nat Commun. 2015 Nov 3;6:7197. doi: 10.1038/ncomms8197 (PMC4659832; doi:10.1038/ncomms8197)
Supplement: Supplementary Information — Supplementary Figures 1-7, Supplementary Table 1 and Supplementary References. [file ncomms8197-s1.pdf]

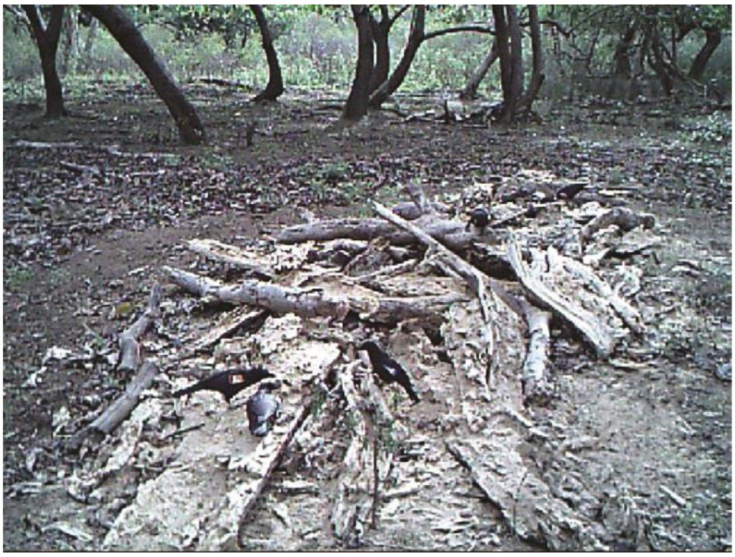

Supplementary Figure 1 Example of an experimental ‘tree-fall’ site. Decaying candlenut wood was transported to the site overnight and ‘salted’ with longhorn beetle larvae, which New Caledonian crows extract from burrows with tools (*cf.* Fig. 1a,b). The image was obtained from the site at the centre of the northern community, using a motion-triggered video camera; three tagged crows are visible in the foreground, while two more are foraging in the background. Due to unreliable motion-triggering, and insufficient resolution to identify crows from their wing-tags, cameras failed to provide useful quantitative data.

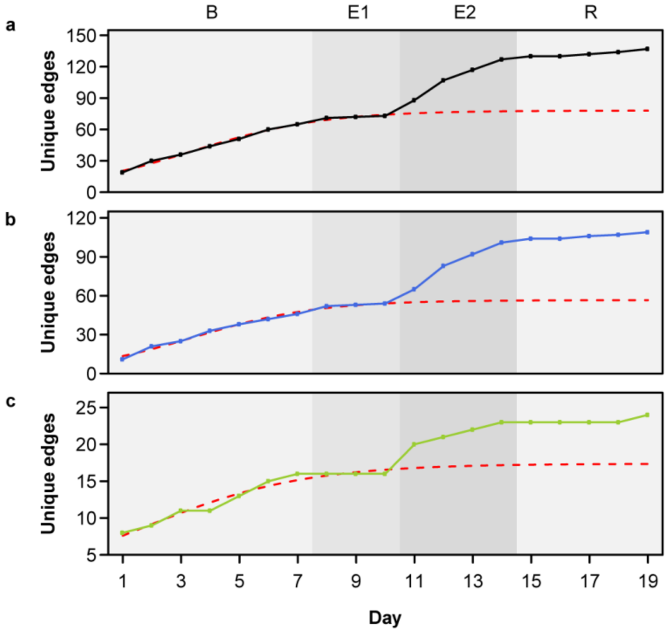

Supplementary Figure 2 Accumulation of novel network edges across study days. Plots show the cumulative total of unique dyadic encounters through the study period: (**a**) for the whole population (black); (**b**) for the northern community only (blue); and (**c**) for the southern community only (green). For illustration purposes, red dashed lines show logistic regression models fitted to the first 10 study days (covering the baseline period, 'B', and experiment 'E1' which failed to elicit a network response; for details, see main text), highlighting the marked impact of the second experiment ('E2') and subsequent slowing during the reversal period ('R'). Note different *y*-axis scales.

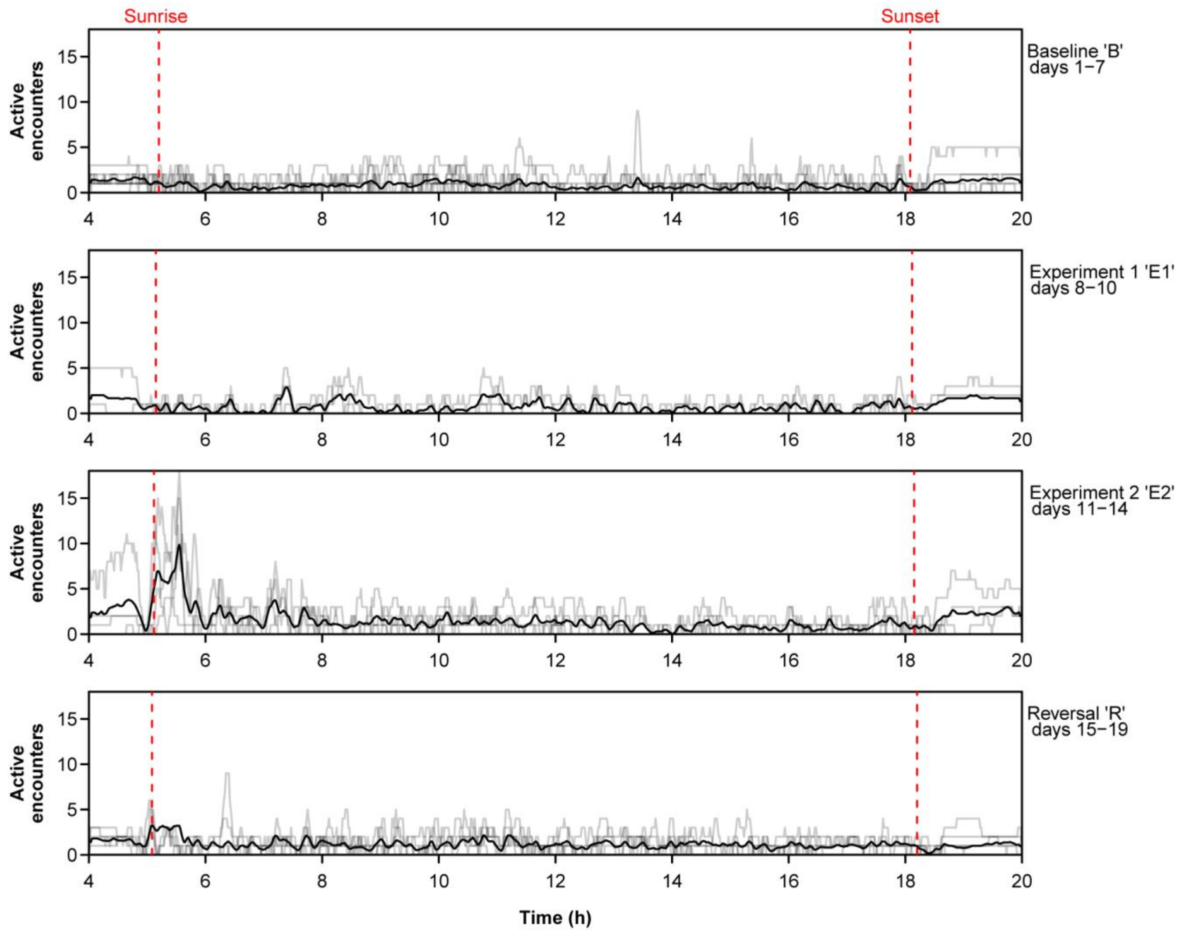

Supplementary Figure 3 Within-day patterns of association according to experimental period. Each grey line shows the number of active (i.e., ongoing) encounters in the population at any given minute. Each black line shows a moving average calculated over a 5-minute window across all days in that experimental period. The panels correspond to the four experimental periods (for details, see main text). Red dashed lines indicate the mean time of astronomical sunrise and sunset for each experimental period.

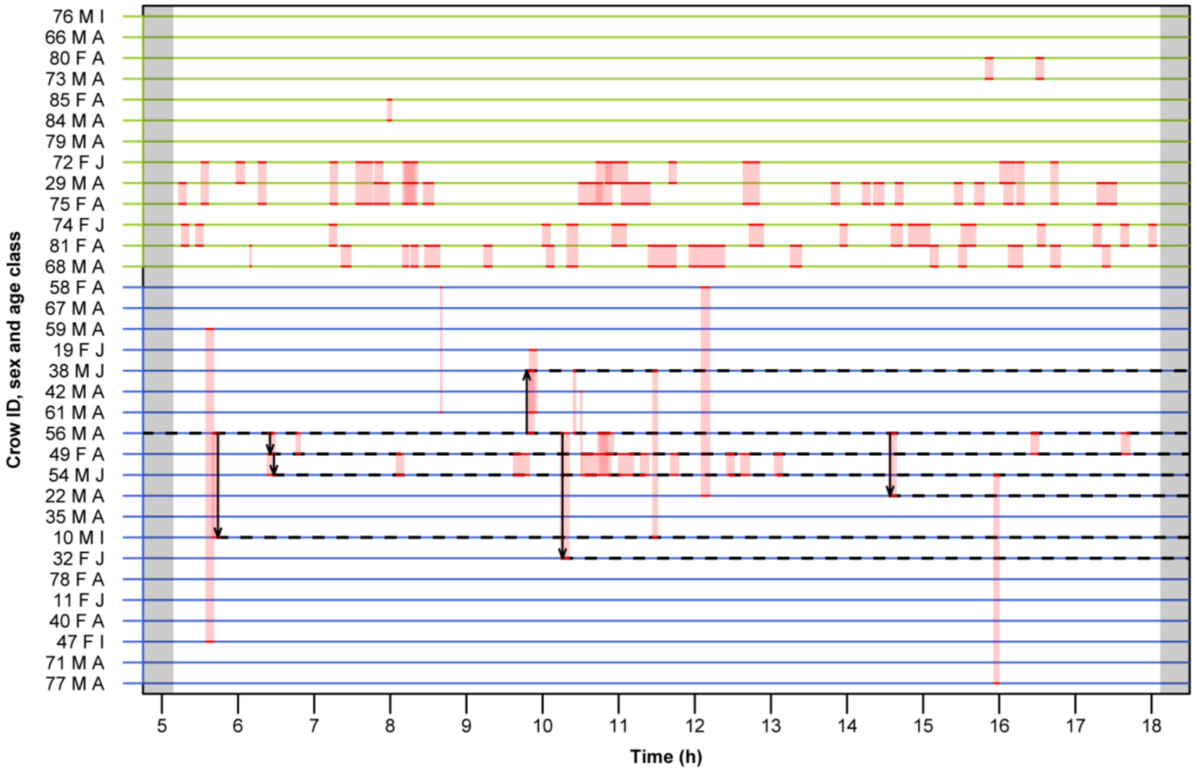

Supplementary Figure 4 Example of actual time-ordered close-range associations among wild New Caledonian crows, with simulated information flow superimposed. Each individual (*n* = 33; labels indicate ID code, sex and age as in Fig. 1d) has a unique timeline, with its association periods shown in red and linked to its social partner(s) by pink shading. Timeline colours indicate community membership (northern community, blue; southern community, green). Instances of information transfer from one crow to another are shown with black vertical arrows (pointing from the source individual to the receiver). A dashed black line is superimposed on the receiver's timeline from the time it receives the information. In this simulation, the information was seeded with crow 56 at the beginning of day 8 (the first day of 'E1'), and had reached six further individuals by the day's end.

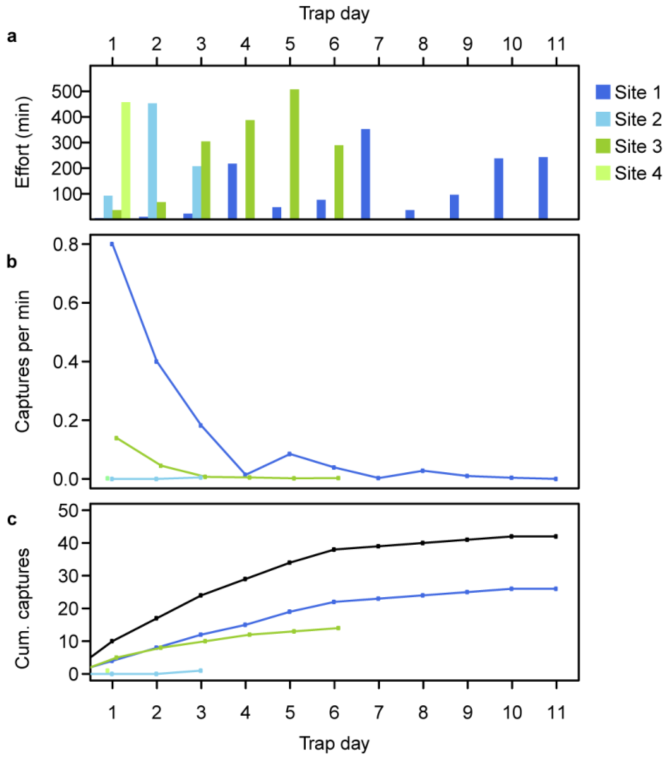

Supplementary Figure 5 Effort and success of crow trapping prior to data collection. (**a**) Time spent trapping at each of four trap sites (for locations, see Fig. 1c). Note that each trap was closed down when no longer productive; all trapping ceased five days before data collection commenced. (**b**) Capture rates (birds per minute) at each trap site. (**c**) Cumulative captures at each trap site (blues refer to trapsites in the northern part of the study area, while greens refer to southern ones) and in total (black line).

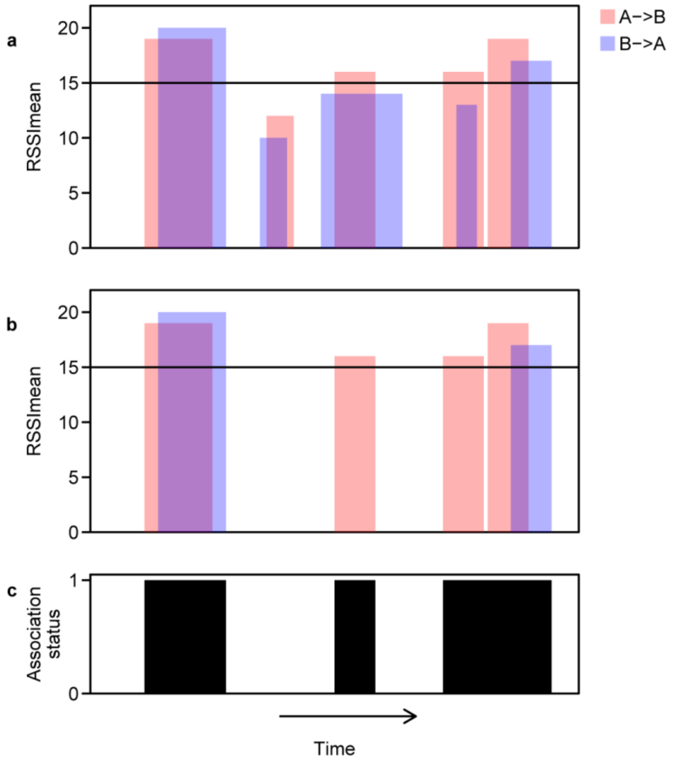

Supplementary Figure 6 Schematic illustration of how tag-recorded encounter logs were filtered and amalgamated. (**a**) Encounters between individuals A and B are logged separately by each bird's transceiver tag. (**b**) Logs below a certain mean signal strength (RSSI*mean*) threshold are removed (unless stated otherwise, in this study RSSI*mean* ≥ 15). (**c**) Logs separated by 23 seconds or less (here shown by the final two A–B logs, in pink) are consolidated. For further details, see ref. 1.

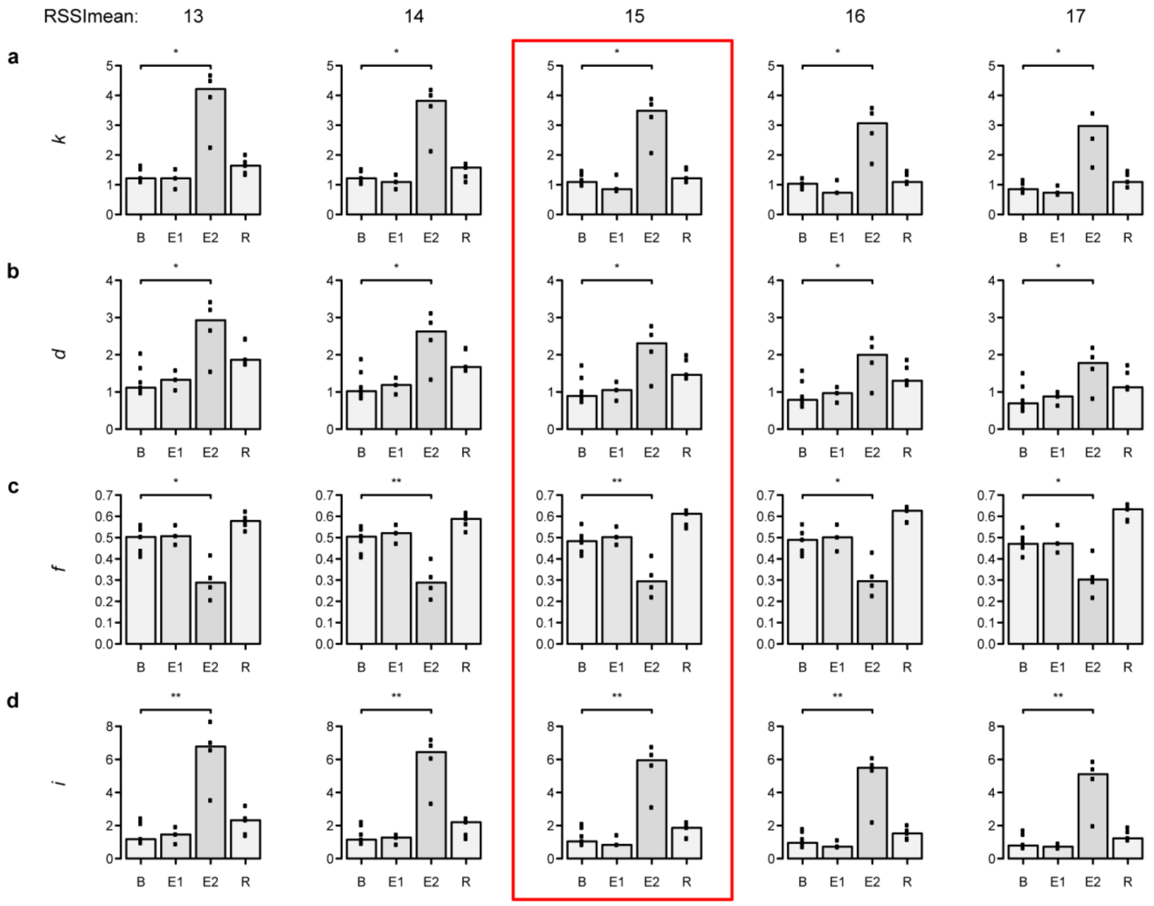

Supplementary Figure 7 Sensitivity of experimental effects to RSSI*mean* filtering value. Each plot shows, according to experimental period (Baseline 'B', Experiment 1 'E1', Experiment 2 'E2', Reversal 'R'), summary statistics of selected metrics (*cf*. Fig. 2) for networks generated using different RSSI*mean* filtering values; points show daily averages, while bars show the median for each experimental period; asterisks indicate significance level of (Wilcoxon rank-sum) comparisons between periods B and E2 (one star, $p < 0.05$; two stars, $p < 0.01$). The results presented in the main text refer to RSSI*mean* ≥ 15 (red box). (**a**) Mean degree (*k*); (**b**) mean duration of summed associations (*d*); (**c**) fraction of *d* spent with first-order kin (*f*); and (**d**) mean daily indomain of diffusion simulations (*i*).

Supplementary Table 1 Attributes of trapped New Caledonian crows and deployed 'Encounternet' tags. Sex denoted by M (male) or F (female). Estimated age class denoted by J (juvenile, < 1 year), I (immature, 1–2 years) or A (adult, > 2 years). Community assignment denoted by N (northern community) or S (southern community).

tag ID	sex	age class	trap site	community assignment	bird mass (g)	tag mass (g)	burden (%)	tag performance
10	M	I	1	N	325	9.88	3.04	minor issues
11	F	J	1	N	235	9.57	4.07	no issues
19	F	J	1	N	235	9.23	3.93	no issues
22	M	A	1	N	330	9.85	2.98	no issues
29	M	A	3	S	330	10.21	3.09	no issues
32	F	J	1	N	255	9.58	3.76	minor issues
35	M	A	1	N	320	9.66	3.02	minor issues
38	M	J	1	N	310	9.75	3.15	no issues
40	F	A	1	N	260	9.36	3.60	no issues
42	M	A	1	N	320	9.89	3.09	no issues
47	F	I	1	N	275	9.59	3.49	minor issues
49	F	A	1	N	255	9.48	3.72	no issues
54	M	J	1	N	300	9.61	3.20	no issues
56	M	A	1	N	330	10.17	3.08	minor issues
58	F	A	1	N	230	9.31	4.05	no issues
59	M	A	1	N	285	9.23	3.24	no issues
61	M	A	1	N	315	9.77	3.10	no issues
66	M	A	3	S	365	9.67	2.65	no issues
67	M	A	1	N	325	10.09	3.10	no issues
68	M	A	3	S	325	9.94	3.06	no issues
71	M	A	1	N	315	9.90	3.14	no issues
72	F	J	3	S	240	9.37	3.90	no issues
73	M	A	3	S	350	9.35	2.67	no issues
74	F	J	3	S	230	9.03	3.93	no issues
75	F	A	3	S	250	9.66	3.86	minor issues
76	M	I	3	S	320	9.31	2.91	no issues
77	M	A	1	N	295	9.37	3.18	minor issues
78	F	A	1	N	260	9.45	3.63	no issues
79	M	A	3	S	345	9.44	2.74	minor issues
80	F	A	3	S	275	9.13	3.32	no issues
81	F	A	3	S	260	9.48	3.65	no issues
84	M	A	3	S	310	9.40	3.03	no issues
85	F	A	4	S	270	9.06	3.36	no issues
17	F	J	1	-	230	9.28	4.03	failed to activate
21	M	A	1	-	300	9.76	3.25	failed on day 11
30	F	I	1	-	245	9.49	3.87	failed to activate
36	F	A	1	-	260	9.54	3.67	failed on day 10
44	M	A	1	-	330	10.16	3.08	failed to activate
45	M	A	1	-	300	9.79	3.26	failed to activate
70	M	J	3	-	290	9.76	3.37	failed on day 4
82	F	I	2	-	275	8.90	3.24	failed on day 7

Reference

1. Rutz, C. *et al.* Calibrating animal-borne proximity loggers. *Methods Ecol. Evol.* DOI: 10.1111/2041-210X.12370 (2015).
